# Revisiting the link between true-self and morality: Replication and extension Registered Report of Newman, Bloom, and Knobe (2014) Studies 1 and 2

**DOI:** 10.1098/rsos.250908

**Published:** 2025-06-25

**Authors:** Shuk Ching Lee, Gilad Feldman

**Affiliations:** ^1^ Department of Psychology, The University of Hong Kong, Hong Kong

**Keywords:** true-self, social psychology, registered report, replication, morality, moral judgements, social norms, essential self

## Abstract

Newman *et al*. 2014 Value judgments and the true self. *Personal. Soc. Psychol. Bull.* **40**, 203–216. (doi:10.1177/0146167213508791) demonstrated that behaviours that are more aligned with moral values are perceived as more strongly reflecting a person’s ‘true-self’, suggesting that morality plays an important role in how people perceive others’ essential self. In this Registered Report, we conducted a close replication of Newman *et al.* 2014 Value judgments and the true self. *Personal. Soc. Psychol. Bull.* **40**, 203–216. (doi:10.1177/0146167213508791)’s Studies 1 and 2 with an online US American sample recruited from Amazon Mechanical Turk using CloudResearch (*N* = 803). We found support for Study 1’s findings that morally positive changes in others are perceived as more reflective of true-self than morally negative changes, in both the forced-choice (original: *η²p* = 0.39, 95% CI [0.25, 0.51]; replication: *η²p* = 0.20, 95% CI [0.16, 0.23]) and the continuous scale (original: *η²p* = 0.33, 95% CI [0.19, 0.45]; replication: *η²p* = 0.22, 95% CI [0.15, 0.25]) measures. We found support for Study 2’s findings that changes more aligned with observers’ political moral views are perceived as more reflective of true-self (original: *η²p* = 0.04, 95% CI [0.00, 0.11]; replication: *η²p* = 0.35, 95% CI [0.29, 0.41]). Extending the replication, we examined associations between true-self attributions and perceived social norms and found that social norms were positively associated with true-self attributions (Study 1: most *r*s ranged from 0.07 to 0.21; Study 2: *r*s = 0.10 to 0.30). Materials, data and analysis code are available on https://doi.org/10.17605/OSF.IO/9FVTQ. This Registered Report has been officially endorsed by Peer Community in Registered Reports: https://doi.org/10.24072/pci.rr.100372.

## 1. Background

True-self is a mental concept that reflects the deepest and most authentic part of a person’s identity, and people tend to evaluate their true-selves as positive. Newman *et al*. [1] proposed that attributions of true-self in others follow a similar pattern. They demonstrated that morally positive changes in others are perceived as more reflective of their true-selves, and that political views guide what people view as morally positive or negative, and so moderate the effect.

In this Registered Report, we conducted a close replication and extension of Newman *et al*. [1] with the following goals. Our first goal was to replicate the associations found between morality and true-self attributions. Our second goal was to add extensions examining (i) true-self attributions associations with perceived social norms, and (ii) lay-beliefs regarding true-self being inherently good or bad, and comparing these for self versus others.

We begin by introducing the literature on the true-self and the chosen article for the replication—Newman *et al*. [1]. We then review the target article and summarize their hypotheses and findings, and then finally present our adjusted design and suggested extensions.

### 1.1. True-self

True-self is defined as the most essential and authentic part of the person’s personality [2–4], whereas surface-self refers to the more superficial aspects of the self in a person [5,6].

There has been increasing interest in the concept of true-self in both the social psychology and experimental philosophy domains [3,7]. One common direction of research has been examining associations between true-self and well-being, such as that the subjective feelings of knowing oneself are associated with increased self-esteem and in meaning of life [8]. Overall, the idea of true-self seems to be linked with positive aspects for the self, and there is a general tendency for people to evaluate the true-self as positive and moral. Morality is perceived as an essential part of true-self ([9]; recent replication by [10]), true-self attributions are influenced by moral judgements [4,11], and people tend to perceive their true-selves as morally good [9,12,13], across ages and cultures [12,14]. This link is helpful in offering some explanations to documented asymmetries in moral judgements [5] and is possibly rooted in psychological essentialism [4,7].

### 1.2. Choice of study for replication: Newman *et al*. (2014)

We conducted an independent well-powered close replication of Newman *et al*. [1]’s Studies 1 and 2, following on the growing recognition of the importance of reproducibility and replicability in psychological science [15,16]. We chose the article by Newman *et al*. [1] based on several factors: its academic impact, the potential in methodological improvements and adjustments, and the suitability of its design for adding extensions that would help gain additional valuable insights.

De Freitas *et al*. [12] conducted a conceptual replication which seems the closest to Newman *et al*. [1], building on their design, examining associations with misanthropy and culture, and reporting a consistent tendency to view the true-self as morally good. A recent conceptual replication by Lefebvre & Krettenauer [17] used a similar design to the target’s Study 1 and concluded that across age groups people do tend to view the true-self as moral. We considered these as evidence in support of the phenomenon, yet saw the potential in stronger evidence with a well-powered direct pre-registered replication to try and obtain more precise estimates of the effect size. The reported effects in their Study 1 were very large and probably over-estimated, and as far as we know their Study 2 examining political views as a moderator has not received as much attention with similar conceptual replication attempts.

The target article has had an impact on scholarly research in social psychology, philosophy, judgement and decision-making, and cognitive science [4,5,11]. At the time of writing (April 2025), there were 356 Google Scholar citations and some important follow-up theoretical and empirical articles, such as Strohminger & Nichols’ [9] work on the essential moral self, recently successfully replicated by Wong & Feldman [10].

### 1.3. Hypotheses and findings in target article

The article by Newman *et al*. [1] consisted of three experiments, and we focused our replication on Studies 1 and 2. We chose these studies given that these were the baseline demonstration and more simplified in their design, and given that Study 3 involved aspects of religion, a topic that is considered more sensitive and fast changing in the US American population.

We combined the two studies into a singular data collection, displayed in random order, and made slight adjustments and added extensions to both studies. This design allowed us to both test the designs of the original studies, and to then run further tests in comparing the effects of the different studies with the potential of additional insights. We successfully employed similar designs in previous replications by our team (e.g. [18–20]).

Their Studies 1 and 2 tested two main hypotheses, summarized in table 1. In their Study 1, the authors hypothesized and demonstrated that others’ morally positive change was more likely than others’ morally negative change to be associated with the true-self. In Study 2, the authors predicted and demonstrated that participants’ own moral values determined true-self attributions such that changes aligned with political views were more likely to be perceived as reflections of true-self. The authors argued that a person’s morality is dependent on one’s own views and values (e.g. [2,21]), which in turn shapes their evaluations of what reflects true-self.

**Table 1.** Replication and extension: hypotheses.

study	hypotheses	description of hypothesis
1	1 (replication)	a morally positive change is perceived as more reflective of true-self than a morally negative change or a morally neutral change
2	2 (replication)	political views moderate the effect, such that change more aligned with liberal values is rated as more reflective of true-self by liberals than conservatives, whereas change more aligned with conservative values is rated as more reflective of true-self by conservatives than liberals
1−2	3a (extension as exploratory)	competing hypothesis: perceived social norms are positively associated with true-self and moral attributions
	3b (extension as exploratory)	competing hypothesis: perceived social norms are negatively associated with true-self and moral attributions

We summarized the findings in the target article in table 2.

**Table 2.** Newman *et al*. [*1*]: summary of findings. CIL = lower bounds CIs. CIH = higher bounds CIs.

study	factors	reported statistics			*η*²	CIL	CIH
		*F*	d.f.	*p*			
1	main effect positive–negative on true-self (forced-choice)	39.92	2127	<0.001	0.39	0.25	0.51
1	main effect positive–negative on true-self (continuous)	31.01	2127	<0.001	0.33	0.19	0.45
2	interaction between political orientation and conservative-liberal on true-self evaluations (continuous)	8.44	1199	= 0.004	0.04	0.00	0.11

### 1.4. Extensions

#### 1.4.1. Study 1: morality valence manipulation check

In the target article, the valence of the moral change was assumed yet never directly tested, and so it is possible that some participants perceived items classified under ‘morally good’ as neutral or even morally bad and ‘morally bad’ items as neutral or even morally good. Furthermore, the target article assumed a clear dichotomy between positive and negative, which greatly simplifies the moral complexity of the items, and limits analyses that consider positive–negative as a continuous scale. We therefore added a morality valence continuous measure as a manipulation check to assess whether participants truly perceive the moral valence of the changes described in the vignettes in the way the experimenters intended, and to allow for testing of associations with other continuous variables.

#### 1.4.2. Study 1: continuous true-self and surface-self measures

The target article forced answers using a dichotomy of true-self versus surface-self. We added continuous measures of true- and surface-selves to try and gain a clearer more comprehensive understanding of the effect and the distinction between the two.

#### 1.4.3. Study 2: vignette political views attribution manipulation check

We added a political views attribution measure as a manipulation check to assess whether participants truly perceive the political view affiliation of the changes described in the Study 2 vignettes in the same way the experimenters intended.

#### 1.4.4. Study 2: capturing diverse political orientations

The target article forced a dichotomy of being either liberal or conservative, and by doing so may have failed to capture more complex political categories, possibly resulting in those who do not think of themselves as being conservative or liberal to identify themselves as belonging to one of the two groups. We expected political orientations to be more diverse than the dichotomy used by the target article and therefore expanded the political views options to also allow participants to indicate if they are ‘independent’ or ‘other’, to try and better capture those who do not self-identify as conservatives or liberals. We thought that this adjustment would probably reduce noise and provide for a more accurate test of the hypotheses.

#### 1.4.5. Study 2: continuous political orientation measure

Political orientations can be more complex than a simple dichotomy contrasting liberals and conservatives, and we therefore supplemented the categorical political orientations measure with a continuous measure between liberal and conservative, allowing for the midpoint option of being politically ‘neutral’. We thought that this adjustment has the potential of better capturing complex political views and therefore to more accurately estimate associations between political orientations and attributions.

#### 1.4.6. Studies 1 and 2: perceived social norms (exploratory)

We aimed to extend the replication study by examining associations between perceived social norms, true-self attributions and morality. The target article’s reference to morality shifted between examining an absolute positive–negative dichotomy in Study 1, where bad was defined and categorized by the experimenters, to examining individuals’ own moral values in Study 2.

Given the hypothesized link between morality and perceptions of true-self, there are two research questions in respect to social norms. The first is regarding whether one’s morality is aligned with perceived social norms, which may bridge between the different perspectives of morality captured in Study 1 (absolute) versus Study 2 (relativistic). The second is regarding whether perceived social norms are associated with perceived true-self: is true-self aligned with perceived social norms? True-self may be perceived stronger when one follows social norms and social construal of morality, yet it is also possible that true-self is perceived stronger when one is perceived as choosing to deviate from social norms and therefore expressing a more free and authentic self separate from others. The link proposed in the target article between morality and true-self implies that adhering to social moral norms and values is associated with stronger perceptions of true authentic self. If that holds true then true-self is seen more in regard to and in alignment with others rather than as differentiating and separate from others. This links with an interesting debate in experimental philosophy and social psychology regarding the purpose of free will [22,23] with two competing views with one viewing free will as meant for ‘following rules’ in overcoming oneself in order to coexist with others in society, and the second viewing free as meant for allowing for pursuit of one’s own wants and needs.

We therefore planned to run an exploratory extension examining associations of morality and true-self perceptions with perceived social norms.

#### 1.4.7. Studies 1 and 2: intuitive true-self belief (exploratory)

The target article conducted an indirect test whether people perceive true-self to be more aligned with morality and good and bad by asking participants to indicate their perceptions regarding described changes in a person’s character. The implicit nature of the target article’s design introduces several challenges. When evaluating true-self by evaluating changes in character, participants might be affected by a variety of factors, such as the feasibility and likelihood of such a change, which may conflict with perceptions of morality which are often considered as an essential stable and durable part of the self [9].

We therefore added an exploratory extension to supplement the indirect test with an explicit continuous measure directly asking participants about their generalized lay-beliefs regarding the true nature of the self as being good or bad. Using this extension we can examine the alignment between the target article’s implicit test and our more explicit test of the core hypotheses.

Furthermore, we were open to the possibility that laypersons perceive true-self as more complex than a simple dichotomy of good versus bad, as it is possible that people perceive the true-self as some mix of both good and bad. We therefore included two separate questions about both good and bad.

In addition, building on a comment by reviewer Dr. Caleb J. Reynolds we examined whether perceptions of true-self vary when they are applied to one’s self and when applied to others, with the possibility of finding self–other asymmetries. We therefore examined true-self lay-beliefs both about one’s own true-self and about the average person’s true-self.

### 1.5. Deviations

We followed the original’s structure of the vignettes, and made slight adjustments to better fit with our target sample and current times. We summarized the deviations in table 3. First, we neutralized gender and ethnicity in all vignettes, including the opening description and forced-choice measure. The original study began every vignette with the following sentence: ‘Imagine an individual named __. __ is different from you in almost every way—he has a different occupation and prefers different things than you’. After the amendment, the adjusted unidentified opening description we used was ‘Imagine someone who is different from you in almost every way—this person has a different occupation and prefers different things than you’. For two specific vignettes like ‘father’ and ‘boyfriend,’ we changed it to ‘parent’ and ‘romantic partner’, respectively. Second, the true-self rating in Study 2 was replaced with a 9-point scale used in Study 1 to maintain consistency across the studies.

**Table 3.** Replication deviations from the original’s methods and design.

study	change in…	original study’s stimuli	deviation	justification
1 and 2	scenarios	‘deadbeat father’ and ‘jerk boyfriend’ ‘Amir lives in a culture that supports terrorism’	‘father-vignette’ replaced ‘parent’; ‘boyfriend-vignette’ replaced ‘romantic partner’ ‘Amir’ was replaced with ‘someone’	addressing possible gender bias and culture bias
1	forced-choice measure	(i) ‘his ‘true-self’ (the deepest, most essential aspect of his being)’, (ii) ‘his ‘surface-self’ (the things that he learned from society or others)’, (iii) ‘none of the above’	(i) ‘the person’s ‘true-self’ (the deepest, most essential aspect of this person’s being)’, (ii) ‘this person’s ‘surface-self’ (the things that this person learned from society or others)’, (iii) ‘none of the above’	addressing possible gender bias
2	true-self rating	a slider bar with ‘strongly disagree’ and ‘strongly agree’ as end points; the corresponding numerical values were 0 and 703	replaced with a 9-point scale with ‘strongly disagree’ and ‘strongly agree’ as endpoints	a more consistent scoring between dependent variables

### 1.6. Pre-registration and open science

We provided all materials, data and code on: https://osf.io/9fvtq/.

This Registered Report was submitted to *Royal Society Open Science* following peer review and recommendation for Stage 2 acceptance at the *Peer Community In* (PCI) *Registered Reports*’ platform. Full details of the peer review and recommendation of the paper at PCI Registered Reports may be found at the links below. After submission to the journal, the paper received no additional external peer review, but was accepted on the basis of the Editor’s recommendation according to the RSOS PCI Registered Reports’ policy (https://royalsocietypublishing.org/rsos/registered-reports#PCIRR). Stage 1 recommendation and review history: https://rr.peercommunityin.org/articles/rec?id=174; https://osf.io/v2tpf/ (our frozen pre-registration version of the entire Stage 1 packet: https://osf.io/k5x4z/). Stage 2 recommendation and review history: Chambers [24]; https://doi.org/10.24072/pci.rr.100372. All measures, manipulations and exclusions conducted for this investigation are reported, and data collection was completed before analyses. The project was part of a large mass replications and extensions project [25], which received ethics approval from the University of Hong Kong (#EA210265). This Registered Report was written based on the Registered Report template by Feldman [26].

## 2. Method

### 2.1. Reproducibility checks

We calculated effect sizes (ES) and power based on the statistics reported in the target article with the help of a guide by Jané *et al*. [27]. We ran into minor challenges in our calculations of the effects reported in Study 2, which reported a two-way interaction comparing conservatives with liberals on conservative versus liberal items. Our calculations suggested minor differences from the values reported in the article. For example, our recalculation of the first post hoc comparisons for conservatives based on the reported *t-*statistic reported resulted in *p* = 0.007; *d* = 0.19, 95% CI [0.01, 0.37] rather than the reported *p* = 0.04, and our recalculations based on the raw descriptives provided (which match with the means in the figure) also seem to result in weaker effects. We consider these rather minor issues, and our current understanding is that these do not change the conclusions of the article. Without access to the raw data and a better understanding of the statistics (correlations between the dependent measures in the mixed models) it is not possible for us to fully deduce the exact effects.

### 2.2. Power analysis and sensitivity analyses

We used the R package ‘pwr’, initially aiming to choose the smallest effect size of the two studies to ensure enough power for all measurements. We provided further information regarding these calculations in the ‘Effect size calculations and power analysis’ subsection in the electronic supplementary material.

The effect sizes reported in Study 1 were very large (*η*² = 0.39, 95% CI [0.25, 0.51]; *η*² = 0.32, 95% CI [0.19,0.45]), and our power analysis indicated a required sample size of 40 (alpha = 0.05, power = 0.95). These were most likely overestimated effects. The effect sizes reported in Study 2 were weaker (*η*² = 0.04, 95% CI [0.00, 11]; Cohen’s *d* = 0.19 [0.01, 0.37]) and our power analysis indicated that the smallest required sample size was 310.

Given the possibility that the original’s effects are overestimated, even in Study 2, we used the suggested Simonsohn [28] rule of thumb, even if meant for other designs, and multiplied 310 by 2.5 resulting in 775 participants, aiming for a total sample of 800 participants. A sensitivity analysis using G*Power [29] indicated that a sample of 800 would allow the detection of *f* = 0.06–0.07 (interaction for: groups = 2, measures = 2/3) and *d* = 0.12 for dependent samples *t*‐test contrasts (both 95% power, alpha = 5%, one-tail), effects weaker than any of the supported effects reported in the target article and the standard effects in social psychology for weak effects [27].

### 2.3. Participants

We recruited a total of 803 US American participants from Amazon Mechanical Turk American using CloudResearch MTurk Toolkit (30; *M*_age_ = 43.18, s.d. = 12.76; 398 females, 393 males, 13 preferred not to disclose/other). We recruited participants with an approval rate between 95% and 100% and the number of tasks approved between 5000 and 100 000. We employed the following CloudResearch MTurk Toolkit options which were considered best practices at the time to ensure high-quality sample: Duplicate IP Block, Suspicious Geocode Block and Verify Worker Country Location, and we recruited only from CloudResearch Approved Participants. We note that 847 subjects began the survey but 44 did not proceed beyond the consent and verifications. We summarized a comparison of study characteristics between the target article and the replication in table 4.

**Table 4.** Comparison between the study characteristics between the original study and the replication.

	Newman *et al*. [1]	replication and extension
sample size	Study 1: 130 Study 2: 201	803
geographic origin	not specified	US American
gender	Study 1: 72% female Study 2: 67% female	393 males, 398 females, 12 other/did not disclose
median age (years)	not specified	41.0
average age (years)	Study 1: 37.0 Study 2: 38.8	43.18
standard deviation age (years)	not specified	12.76
age range (years)	not specified	21−91
medium (location)	computer (online)	computer (online)
compensation	gift certificates	USD 1.9
year	2014	2022

### 2.4. Design and procedure

We reached out to the authors of the target article and are grateful for the materials they provided, which were helpful in our reconstruction of the materials. They have also been responsive and supportive in follow-up interactions. We followed the experimental designs by the target article and summarized the two studies in tables 5 and 6.

**Table 5.** Study 1: summary of experimental design. IV = independent variables. DV = dependent variables.

IV1: block 1 order mix (between-subject)	IV1: block 2 order mix (between-subject)
positive change (subset b) + negative change (subset a) + neutral	positive change (subset a) + negative change (subset b) + neutral (same)
(same) (valence within-subject):	(valence within-subject):
alcoholism-positive change boss-positive change parent-positive change ethnic minorities-positive change terrorism-negative change business practices-negative change romantic partner-negative change police officer-negative change Mac computer-neutral change country-neutral change cat-neutral change football-neutral change	alcoholism-negative change boss-negative change parent-negative change ethnic minorities-negative change terrorism-positive change business practices-positive change romantic partner-positive change police officer-positive change PC computer-neutral change city-neutral change dog-neutral change baseball-neutral change
**true-self rating** DV forced-choice (replication) please rate what aspect of the person’s personality caused the described change on a choice between: (1) this person’s true-self, (2) this person’s surface-self and (3) none of the above. DV continuous (replication) please rate to what extent this person is being true to the deepest, most essential aspects of their being. 0 = *not at all true to oneself*; 9 = *very much true to oneself*. **true-self measure** DV continuous (extension) please rate the extent to which the change is a reflection of true-self 0 = *not at all* to 100 = *completely.* please rate the extent to which the change is a reflection of surface-self 0 = *not at all* to 100 = *completely.*	
**DV: morality valence** (extension manipulation check) do you perceive this person’s change as morally good or morally bad? −100 = *very* bad; 0 = *neither*; 100 = *very good*	
**DV social norms** (exploratory extension) please rate to what extent the described change is in line with the social norms on a scale of −100 to 100 (*very much against social norms* to *very much in line with social norms*).	
preferences towards neutral items in experiment 1 (replication) (presented at end of Study 1) please indicate your own personal preferences on a 5-point scale with, for example, ‘strongly prefer dogs’ and ‘strongly prefer cats’ as endpoint and ‘no preference’ as the midpoint.	
explicit measures of true-self intuitions (exploratory extension) (presented at the end after both studies completed) (shared with study 2). See ‘extensions’ under §1.4.7’	

**Table 6.** Study 2: summary of experimental design. IV = Independent variables. DV = dependent variables.

IV: condition (within-subject)
moral changes in terms of different political orientations conservative direction changes (within): homosexuality change patriotism change theism change monogamy change liberal direction changes (within): global warming change gender equality change helping others change abortion change
**DV: true-self rating** (replication) please rate to what extent at this person’s very essence, there was always something deep within them calling them to___ , and then this true-self emerged 0 = ‘*strongly disagree*’; 9 = ‘*strongly agree*’.
**DV: political orientation** (extension manipulation check) do you perceive this person’s change as liberal or conservative? −100 = *pro-conservative*; 0 = *neither*; 100 = *pro-liberal.*
**extension DV: social norm** (exploratory extension) please rate to what extent the described change is in line with the social norms −100 = ‘*very much against social norms*’ to 100 = ‘*very much in line with social norms*’.
explicit measures of true-self intuitions (exploratory extension) (presented at the end after both studies completed) (shared with Study 1). See ‘extensions’ under §1.4.7.
(presented after both studies completed) categorical political measure (adjusted replication) please choose the one that you feel best represents your political views. ‘*conservative*’, ‘*liberal*’, ‘*independent*’ and ‘*other*’. (‘independent’ and ‘other’ are adjustments). continuous political measure (extension) ‘please indicate your political orientation along the conservative-liberal scale’ 1 = ‘*extremely conservative*’; 4 = ‘*centre*’; 7 = ‘*extremely liberal*’.

We ran the two studies together in a single data collection, with the display of scenarios and conditions counterbalanced using the randomizer ‘evenly present’ function in Qualtrics. Scenarios were presented in random order and participants were randomly and evenly assigned into the different conditions. This unified design combining replications of several studies into a singular data collection was previously tested successfully in many of the replications and extensions conducted by our team (e.g. [18–20]), and is especially powerful in addressing concerns about the target sample (naivety, attentiveness, etc.) when some studies replicate successfully whereas others do not, as well as in the potential in drawing inferences about the links between the different studies and consistency in participants’ responding to similar paradigms.

Participants first read a consent form and indicated their willingness to participate, and then answered several verification questions. Participants first indicated their consent, with three questions confirming their eligibility, understanding and agreement with study terms, which they had to answer with a ‘yes’ and the required responses in order to proceed to the study. The three questions also served as attention checks, with a randomized display order of the options—(i) ‘Are you able to pay close attention to the details provided and carefully answer questions that follow?’ (yes/no/not sure), (ii) ‘Do you understand the study outline and are willing to participate in a survey with comprehension checks?’ (yes/no/not sure), and (iii) ‘Are you a native English speaker born, raised, and currently located in the US?’ (yes/no). Failing any of the three questions meant that the participants did not indicate consent and therefore could not embark on the study. Upon completion of these steps, participants proceeded to begin the survey.

Participants answered the replications of the Studies 1 and 2 in random order. Participants rated true-self attributions regarding moralized changes (Study 1: generalized good versus bad changes; Study 2: moral changes aligned with liberal versus conservative values).

In line with the target article’s design, vignettes in Study 1 were prefixed with a matching of moralized changes in each block so that each block had half negative and half positive changes. We thought this design to be suboptimal compared with a more comprehensive randomization, given that it contrasts specific moral changes against one another, yet decided to follow the target’s design as is.

After completing both experiments, participants rated their political views (used in the replication of Study 2) and their generalized lay-beliefs regarding true-self as inherently good and inherently bad (extension). Finally, participants answered funnelling questions and provided demographic information, also indicating their level of English understanding of the survey (1 = *very bad*; 7 = *very good*), and seriousness in answering the survey (1 = *not at all*; 5 = *very much*).

### 2.5. Measures

#### 2.5.1. Replication

With the materials sent by the original authors, we were able to reproduce most of the materials in the study. Stimuli for this replication consisted of 12 vignettes from Study 1 and eight vignettes from Study 2. The opening description for each vignette was ‘Imagine someone who is different from you in almost every way—this person has a different occupation and prefers different things than you’.

Each vignette followed the structure that the person used to engage in behaviour/belief X and is now involved in behaviour/belief Y. In Study 1, changes were framed as good, bad or neutral. A morally good change was framed such that a behaviour/belief changed for the better; a morally bad change was framed such that a behaviour/belief changed for the worse. The direction of change was counterbalanced between conditions. Four were changes the authors categorized as morally good, four as morally bad and four as neutral, and the two exact combinations are provided in table 5. In Study 2, changes were framed as more aligned with either the conservative or the liberal political views. We followed the original study in classifying the vignettes into binary political ideology: four change vignettes were meant as aligned with conservative views (homosexuality to heterosexuality, unpatriotic to patriotic, atheist to religious, promiscuous to monogamous) and four change vignettes were meant as aligned with liberal views (deny global warming to supporting the environment, sexist to egalitarian, greedy to generous and vandalizing abortion clinics to not vandalizing abortion clinics).

##### 2.5.1.1. Study 1

*True-self: forced-choice measure (replication)*

Participants indicated their perceptions of whether the change reflected true-self with three forced-choice options: (i) ‘true-self’ (the deepest, most essential aspect of this person’s being), (ii) ‘surface-self’ (the things that this person learned from society or others), (iii) ‘none of the above’ (with a text entry option).

*True-self: continuous measure—rating after change (replication)*

In Study 1, at the end of each of the 12 vignettes, participants rated whether the person’s final state after the change reflected the person’s true-self (1 = *not at all*; 9 = *very much*).

*Neutral preferences*

Preferences on the four neutral items were evaluated on a 5-point scale with, for instance, ‘*strongly prefer dogs*’ and ‘*strongly prefer cats*’ as the endpoints and ‘*no preference*’ as the midpoint.

##### 2.5.1.2. Study 2

*Continuous true-self rating (replication)*

In Study 2, there was a similar question for each of the eight vignettes with a slight change in describing changes as ‘the extent to which the change resulted from the emergence of the person’s true-self’ (1 = *strongly disagree*; 9 = *strongly agree*).

*Categorical political orientation measure (replication and extension)*

We followed the binary political orientation measure in the original study with an extension adjustment of adding two more choices of ‘other’ and ‘independent’.

### 2.5.2. Extensions

#### 2.5.2.1. Study 1: morality valence manipulation check

In Study 1, we added a manipulation check immediately after the moralized vignettes to assess whether participants assessed the change on a scale from ‘*morally bad*’ (−100) to ‘*neither*’ (0) to ‘*morally good*’ (100).

#### 2.5.2.2. Study 1: continuous true-self and surface-self measures

Participants responded to what extent to which the change reflects true-self and surface-self on two separate scales from 0 (*not at all*) to 100 (*completely*). Participants answered both scales. This was meant to test both surface- and true-self separately and as continuous measures.

#### 2.5.2.3. Study 2: vignette political view manipulation check

In Study 2, we added a manipulation check immediately after the vignettes to examine how participants assessed the changes described in the vignettes: ‘Do you perceive this person’s change as more pro-liberal or more pro-conservative?’ (−100 = *pro-conservative*; 0 = neither; 100 = *pro-liberal*).

#### 2.5.2.4. Study 2: continuous political orientation measure

In addition to the categorical political orientation measure, we added a 7-point continuous measure of political orientation (1 = *extremely conservative*; 4 = *centre*; 7 = *extremely liberal*).

#### 2.5.2.5. Studies 1 and 2: perceived social norms (exploratory)

For all vignettes, participants were asked the degree to which the described change of the person was in line with social norms. Participants responded using a −100 to 100 scale with ‘very much against social norms’ and ‘very much in line with social norms’ as endpoints.

#### 2.5.2.6. Studies 1 and 2: intuitive true-self beliefs (exploratory)

Participants were asked about their lay-beliefs regarding the nature of true-self on a scale of 0 (*not at all*) to 100 (*completely*) on two statements : ‘true-self is morally good’ and ‘true-self is morally bad’. Participants answered these twice (four items overall), once rating their own true-self (‘Please rate your intuitive beliefs regarding your own true-self (the deepest and most essential part)’—‘my true-self is morally good/bad’) and another rating the average person’s true-self (‘Please rate your intuitive beliefs regarding the average person’s true-self (the deepest and most essential part)’—‘average person’s true-self is morally good/bad’).

### 2.6. Evaluation criteria for replication findings

We aimed to compare the replication effects with the original effects in the target article using the criteria set by LeBel *et al*. [31] (see section ‘Replication evaluation’ in the electronic supplementary material).

### 2.7. Replication closeness evaluation

We provided details on the classification of the replication using the criteria by LeBel *et al*. [31] in table 7. We summarize the replication as a ‘close’ replication.

**Table 7.** Classification of the replication based on LeBel *et al*. [31].

design facet	replication	details of deviation
effect/hypothesis	same	
IV construct	same	
DV construct	same	
IV operationalization	same	
DV operationalization	same	
population (e.g. age)	similar	data collected using a sample from MTurk using CloudResearch
IV stimuli	different	neutralized items^a^
DV stimuli	similar	neutralized items and standardized scorings^a^
procedural details	similar	combined Studies 1 and 2, random order
physical settings	similar	online
contextual variables	similar/different	
replication classification	close replication	

^a^ Further details of our deviations can be found in table 3. IV represents independent variable. DV represents dependent variable.

### 2.8. Exclusion criteria

We focused our analyses on the full sample of all participants who completed the study. We had planned to report analyses with exclusions if we failed to find support for the hypotheses (our planned exclusions were (i) participants indicating a low proficiency of English (self-report less than 5, on a 1−7 scale), and (ii) participants who self-report not being serious about filling in the survey (self-report less than 4, on a 1−5 scale). Given that we found support for all the hypotheses, we follow the pre-registered Stage 1 plan and do not report additional analyses with exclusions. As an additional exploratory analysis, with our code on the Open Science Framework (OSF), we also provided the results of our analyses with applying the exclusions, and these had no impact on our findings.

## 3. Results

### 3.1. Replication

We summarized all descriptive statistics in tables 8 and 9, and statistical test results in tables 10 and 11. Plots were created using the JAMOVI [33] jmv R package.

**Table 8.** Study 1: descriptives of true-self rating for moralized change (replication + extension).

conditions		block 1 (*n* = 408) *M* (s.d.)	block 2 (*n* = 395) *M* (s.d.)	overall (*n* = 803) *M* (s.d.)
*replication:* forced-choice measure				
good change		2.47 (1.28)	2.97 (1.17)	2.72 (1.25)
bad change		2.16 (1.41)	2.13 (1.39)	2.14 (1.40)
neutral change		1.64 (1.24)	1.27 (1.32)	1.46 (1.29)
*replication:* continuous true-self rating				
good change		6.55 (1.40)	7.08 (1.29)	6.81 (1.37)
bad change		5.36 (2.02)	5.10 (1.90)	5.23 (1.96)
neutral change		5.83 (1.21)	5.64 (1.15)	5.74 (1.18)
*extension:* continuous true-self and surface-self measures				
good change	true-self surface-self	64.8 (20.6) 47.3 (23.6)	72.1 (19.3) 47.5 (23.6)	68.4 (20.3) 47.4 (23.6)
bad change	true-self surface-self	56.3 (25.2) 47.7 (24.5)	53.5 (24.4) 47.5 (23.6)	54.9 (24.8) 47.6 (24.0)
neutral change	true-self surface-self	48.0 (21.5) 52.8 (21.5)	43.2 (22.3) 55.8 (21.9)	45.7 (22.0) 54.3 (21.8)

Note. *M* indicates mean. s.d. indicates standard deviation. *n*/*N* indicates sample size. There were four items for positive, four for negative, the calculation for ‘forced-choice measure’ is the number of items out of the four that the participant indicated are a reflection of true-self, therefore range is 0−4.

**Table 9.** Study 2: descriptive statistics for true-self attribution on changes favouring liberal and conservative values.

condition	orientation	*n/N*	mean	standard deviation
overall true-self rating (liberal items)		803	5.88	1.39
overall true-self rating (conservative items)		803	5.51	1.58
dichotomy political orientation (replication)				
true-self rating (liberal items)	liberal	414	6.11	1.39
	conservative	218	5.57	1.32
	independent	160	5.75	1.39
	other	11	5.39	1.24
true-self rating (conservative items)	liberal	414	4.89	1.48
	conservative	218	6.63	1.33
	independent	160	5.64	1.26
	other	11	5.11	1.79
continuous political orientation (extension)				
true-self rating (liberal items)	extremely conservative	46	5.54	1.59
	very conservative	96	5.57	1.30
	somewhat conservative	101	5.58	1.23
	centre	122	5.81	1.36
	somewhat liberal	145	6.02	1.32
	very liberal	190	6.05	1.38
	extremely liberal	103	6.22	1.58
true-self rating (conservative items)	extremely conservative	46	7.03	1.21
	very conservative	96	6.81	1.22
	somewhat conservative	101	6.09	1.31
	centre	122	5.65	1.24
	somewhat liberal	145	5.30	1.32
	very liberal	190	4.89	1.51
	extremely liberal	103	4.34	1.53

Note. Mean and standard deviation refer to the descriptive statistics of true-self rating on different political vignettes using categorical scale and continuous scale in Study 2.

**Table 10.** Studies 1 and 2: summary of statistical tests.

study	factor	projects	*F*	d.f.	*p*	*η²p*	CIL	CIH	interpretation
replication									
1	main effect positive–negative on true-self (forced-choice)	original	39.92	2127	<0.001	0.39	.025	0.51	signal, inconsistent, smaller
		replication	199.6	21602	<0.001	0.20	0.16	0.23	
1	main effect positive–negative on true-self (continuous rating)	original	31.01	2127	<0.001	0.33	0.19	0.45	signal, inconsistent, smaller
		replication	223.7	21602	<0.001	0.22	0.15	0.25	
2	interaction between dichotomy political orientation (liberal and conservative) and item types (liberal and conservative) on continuous true-self rating	original	8.44	1199	= 0.004	.004	0.00	0.11	signal, inconsistent, larger
		replication	340.93	1630	<0.001	0.35	0.29	0.41	
extension									
1	main effect positive–negative on true-self (continuous true-self and surface-self measure)		240.5	21602	<0.001	0.23	0.19	0.27	signal
1	main effect positive–negative on surface-self (continuous true-self and surface-self measure)		36.93	21602	<0.001	0.04	0.03	0.06	signal
2	interaction between continuous political orientation and item types (liberal and conservative) on continuous true-self rating		260.9	1801	<0.001	0.34	0.28	0.39	signal

Note. The interpretation of the replication outcomes was based on LeBel *et al*. [32] (see section ‘Additional tables and figures’ in the electronic supplementary material).

**Table 11.** Studies 1 and 2: post hoc tests effect size*.*

study	post hoc tests	original Cohen’s *d*, 95% CI	replication Cohen’s *d*, 95% CI	interpretation
replication				
1	independent sample *t*‐test—forced-choice items (good change versus bad change)	0.53 [0.34, 0.71]	0.62 [0.47, 0.76]	signal, consistent
1	independent sample *t*‐test—true-self rating (good change versus bad change)	0.56 [0.38, 0.75]	1.24 [1.08, 1.39]	signal, inconsistent, larger
2	dependent sample *t*‐test—liberal participants (conservative items versus liberal items)	0.19 [0.01, 0.37]	0.83 [0.72, 0.94]	signal, inconsistent, larger
2	dependent sample *t*‐test—conservative participants (conservative items versus liberal items)	0.31 [0.09, 0.54]	0.72 [0.57, 0.87]	signal, inconsistent, larger
extension				
1	independent sample *t*‐test—continuous true-self measure (good versus bad)	N/A	0.87, [0.72, 1.01]	signal
1	independent sample *t*‐test—continuous surface-self measure (good versus bad)	N/A	−0.02, [−0.16, 0.12]	no signal

#### 3.1.1. Study 1: true versus surface-self: forced-choice item (replication)

**Figure 1.**
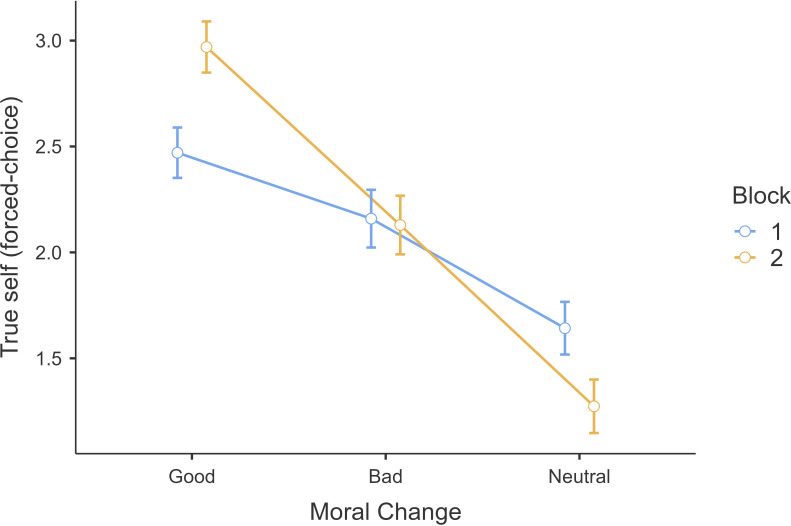
Study 1: forced-choice measure for positive–negative vignettes. Note. True-self forced-choice measure across moral-valenced vignettes of good, bad and neutral between block 1 and block 2.

We conducted a 3 (moral valence vignettes: good, bad, neutral; within) × 2 (order: block 1 and block 2; between) repeated-measure ANOVA and found support for a main effect of moral valence (*F*(2, 1602) = 199.6, *p* < 0.001; *η²p* = .20, 95% CI [0.16, 0.23]), with no main effect for block type, yet support for an interaction with effects being stronger in block 2 (we reported results of the interaction and block type in the ‘Additional tables and figures’ section of electronic supplementary material). Using the aggregate of the two blocks to mirror the original’s analysis, participants were more likely to rate higher true-self for good changes (*M* = 2.72, s.d. = 1.25) than for bad changes (*M* = 2.14, s.d. = 1.40; *t*(801) = 8.72, *p* < 0.001) (figure 1), and neutral changes (*M =* 1.46, s.d. = 1.29; *t*(801) = 20.49, *p* < 0.001; compared with bad changes: *t*(801) = 11.07, *p* < 0.001). We concluded support for Hypothesis 1 that morally good change is more likely to reveal the true-self than morally bad or neutral changes.

Following the original study’s analyses, we performed a chi-squared analysis for all the vignettes separately. We summarized our analyses in table 12. We found support for participants rating the agent’s true-self as more reflected in good changes rather than bad changes among almost all the moralized behaviours, except for the item romantic partner which is consistent with the target article.

**Table 12.** Study 1: chi-squared analysis and independent *t*‐test of forced-choice measure across all vignettes*.*

	good change		bad change				comparing good with bad on true-self			comparing good with bad on surface-self		
moralized items	true-self	surface-self	true-self	surface-self	*X* ^2^	*p*	*t*	*p*	Cohen’s *d* [LCI, HCI]	*t*	*p*	Cohen’s *d* [LCI, HCI]
police officer	311	78	206	196	72.0	<0.001	−8.73	<0.001	−0.62 [−0.76, −0.47]	8.85	<0.001	0.63 [0.48, 0.77]
businessman	307	83	210	192	61.3	<0.001	−8.07	<0.001	−.57 [−.71, −.43]	8.08	<0.001	0.57 [0.43, 0.71]
ethnic minorities	269	130	220	168	9.80	0.007	2.98	0.003	0.21 [0.07,.035]	−3.14	0.002	−0.22 [−0.36, −0.08]
alcoholism	267	133	139	238	73.7	<0.001	8.98	<0.001	0.63 [0.49, 0.78]	−8.17	<0.001	−0.58 [−0.72, −0.43]
terrorism	305	88	189	210	81.5	<0.001	−9.47	<0.001	−0.67 [−0.81, −0.52]	8.97	<0.001	0.63 [0.49, 0.78]
parent	292	111	249	131	9.86	0.007	2.59	0.010	0.18 [0.04, 0.32]	−1.84	0.066	−0.13 [−0.27, 0.01]
boss	180	224	233	152	23.0	<0.001	−4.26	<0.001	−0.30 [−0.44, −0.16]	4.72	<0.001	0.33 [0.19, 0.47]
romantic partner	250	136	276	119	2.94	0.230	1.30	0.195	0.09 [−0.05, 0.23]	−1.60	0.110	−0.11 [−0.25, 0.03]

neutral items	behaviour block 1 (left)		behaviour block 2 (right)				comparing behaviour left with right on true-self			comparing behaviour left with right on surface-self		
Mac/PC	71	305	72	284	1.24	0.539	−0.31	.760	−0.02 [−0.16, 0.12]	0.92	0.361	0.06 [−0.07, 0.20]
country/city	246	139	130	243	63.9	<0.001	8.07	<0.001	0.57 [0.43, 0.71]	−8.09	<0.001	−0.57 [−0.71, −0.42]
cat/dog	230	143	185	176	8.10	0.02	2.71	0.007	0.19 [0.05, 0.33]	−2.76	0.006	−0.20 [−0.33, −0.06]
football/ baseball	123	258	116	253	0.06	0.969	0.24	0.809	0.02 [−0.12, 0.16]	−0.24	0.811	−0.02 [−0.16, 0.12]

Note. X^2^ compares the proportions of true–surface self rating in good versus bad. ‘Behaviour block 1 (left)’ and ‘behaviour block 2 (right)’ refers to sets of neutral items, where block refers to the block of display and right/left refer to which of the pair is displayed. For example, in the Mac/PC pair, Mac = behaviour block 1 (left), PC = behaviour block 2 (right).

We conducted binomial tests comparing the frequency of true-self and surface-self choices within each vignette to a random 50%–50% split. Consistent with the findings above, we found support for the difference in true-self rating for all vignettes compared with the surface-self rating in each vignette. Across all morally good vignettes, there were more ‘true-self’ choices than ‘surface-self’ responses. In comparison, in the morally bad vignettes, only four vignettes indicated more ‘true-self’ than ‘surface-self’ choices, one indicated more ‘surface-self’ choices than ‘true-self’, with the other three showing no support for differences from a 50−50 random split. In the neutral vignettes, five out of eight had more ‘surface-self’ than ‘true-self’ responses. In general, participants were more likely to attribute true-self in good changes than bad changes, except romantic partners, though bad changes had more ‘true-self’ than ‘surface-self’ responses than we initially expected. We provided more details in the ‘Additional tables and figures’ subsection of the electronic supplementary material.

#### 3.1.2. Study 1: continuous true-self after change rating (replication)

**Figure 2.**
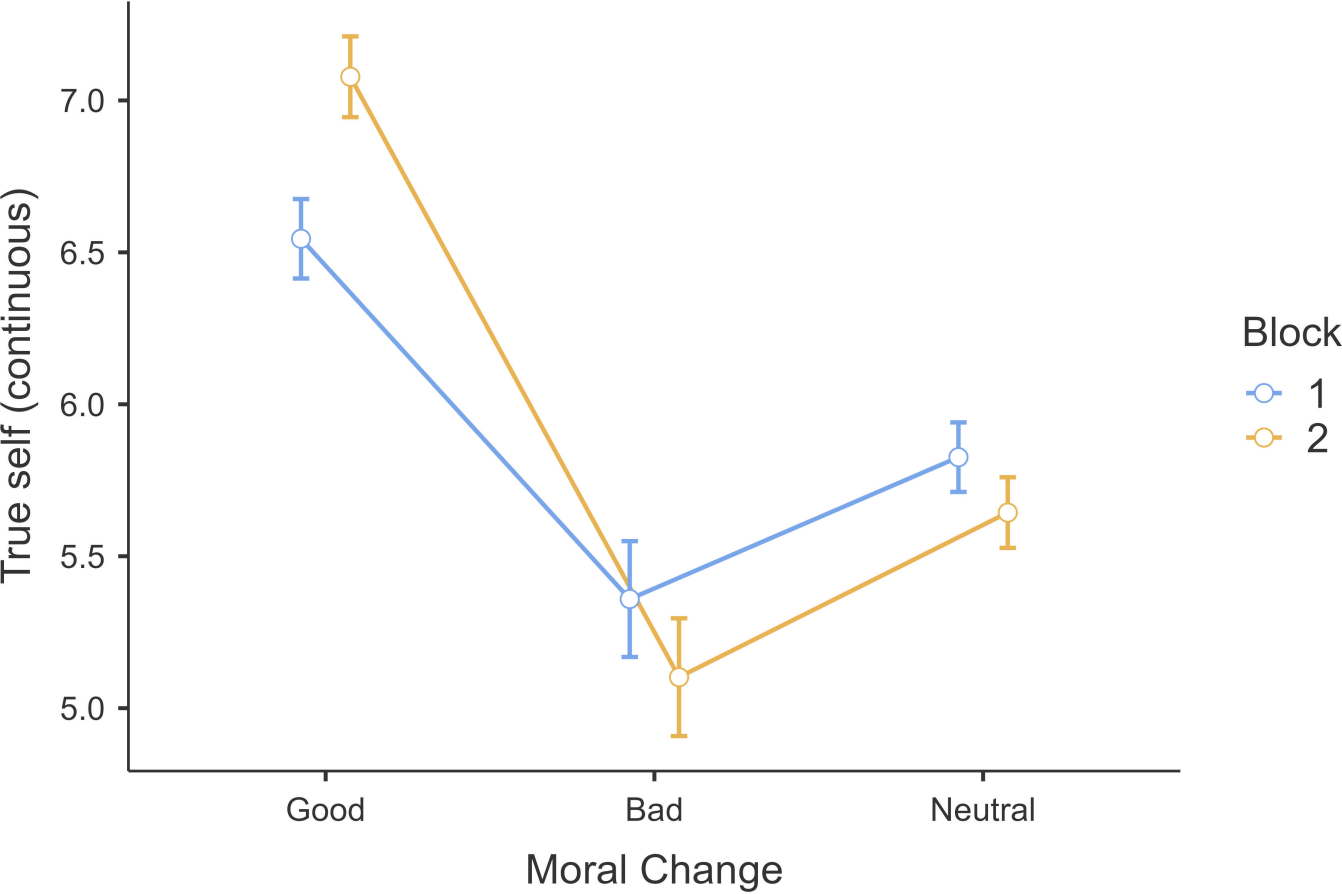
Study 1: continuous true-self ratings measure for positive–negative vignettes. Note. Continuous true-self rating across moral-valenced vignettes of good, bad and neutral between block 1 and block 2.

Similarly, we conducted a 3 (moral valence: good, bad, neutral; within) × 2 (order: block 1 and block 2; between) mixed-model ANOVA on the continuous true-self after change ratings and found support for a main effect for moral valence (*F*(2,1602) = 223.7, *p* < 0.001, *η*²*p* = 0.22, 95% CI [0.15, 0.25]), no support for a main effect for block type effect, and with support for an interaction (we reported results of the interaction effect and block type effect in the ‘Additional tables and figures’ of electronic supplementary material). We found support for differences between true-self ratings for good change (*M =* 6.81, s.d. *=* 1.37) versus bad change (*M =* 5.23, s.d. *=* 1.96, *t*(801) = 17.50, *p <* 0.001) (figure 2) and compared with the neutral change (*M* = 5.74, s.d. = 1.18; *t*(801) = 19.08, *p <* 0.001). We again found support for Hypothesis 1 that morally good change is more reflective of true-self using the continuous measure.

We summarized a series of *t*-tests comparing the true-self rating on the moral-valence behaviours for all vignettes in table 13. For all moralized vignettes, we consistently found support that participants were more likely to agree that morally good behaviour reflected the agent’s true-self than the morally bad behaviour. We failed to find support for any difference between the neutral vignettes, except the ‘country/city’ item.

**Table 13.** Study 1: independent *t*‐test comparing positive–negative on continuous true-self ratings.

moralized items	*t*	*p*	Cohen’s *d* [LCI, HCI]
police officer	13.3	<0.001	.94 [−1.09, −0.79]
businessman	12.4	<0.001	.88 [−1.03, −0.73]
ethnic minorities	8.00	<0.001	.56 [0.42, 0.71]
alcoholism	16.3	<0.001	1.15 [0.99, 1.31]
terrorism	14.2	<0.001	1.01 [−1.16, −0.85]
parent	10.2	<0.001	0.72 [0.57, 0.86]
boss	2.79	0.005	0.20 [0.06, 0.34]
romantic partner	3.96	<0.001	0.28 [−0.42, −0.14]

neutral items	*t*	*p*	Cohen’s *d* [LCI, HCI]
Mac/PC	−1.19	0.233	−0.08 [−0.22, 0.05]
country/city	6.38	<0.001	0.45 [0.31, 0.59]
cat/dog	2.12	0.034	0.15 [0.01, 0.29]
football/ baseball	−1.51	0.132	−0.11 [−0.25, 0.03]

Note. In the moralized items, true-self ratings for positive change were always higher than true-self ratings for negative change. In the neutral items, the higher true-self ratings were for PC, country, cat and baseball.

#### 3.1.3. Study 2: interaction between political orientation and political item type on true-self rating (replication)

We conducted a 2 (political view: liberal and conservative; between) × 2 (item types: liberal and conservative; within) mixed-model ANOVA and found support for an interaction (*F* (1,630) *=* 340.93, *p <* 0.001, *η*²*p* = 0.35, 95% CI [.29, .41]) and political view main effect, but no item type main effect (additional details are provided in the subsection under ‘Additional tables and figures’ of electronic supplementary material).

**Figure 3.**
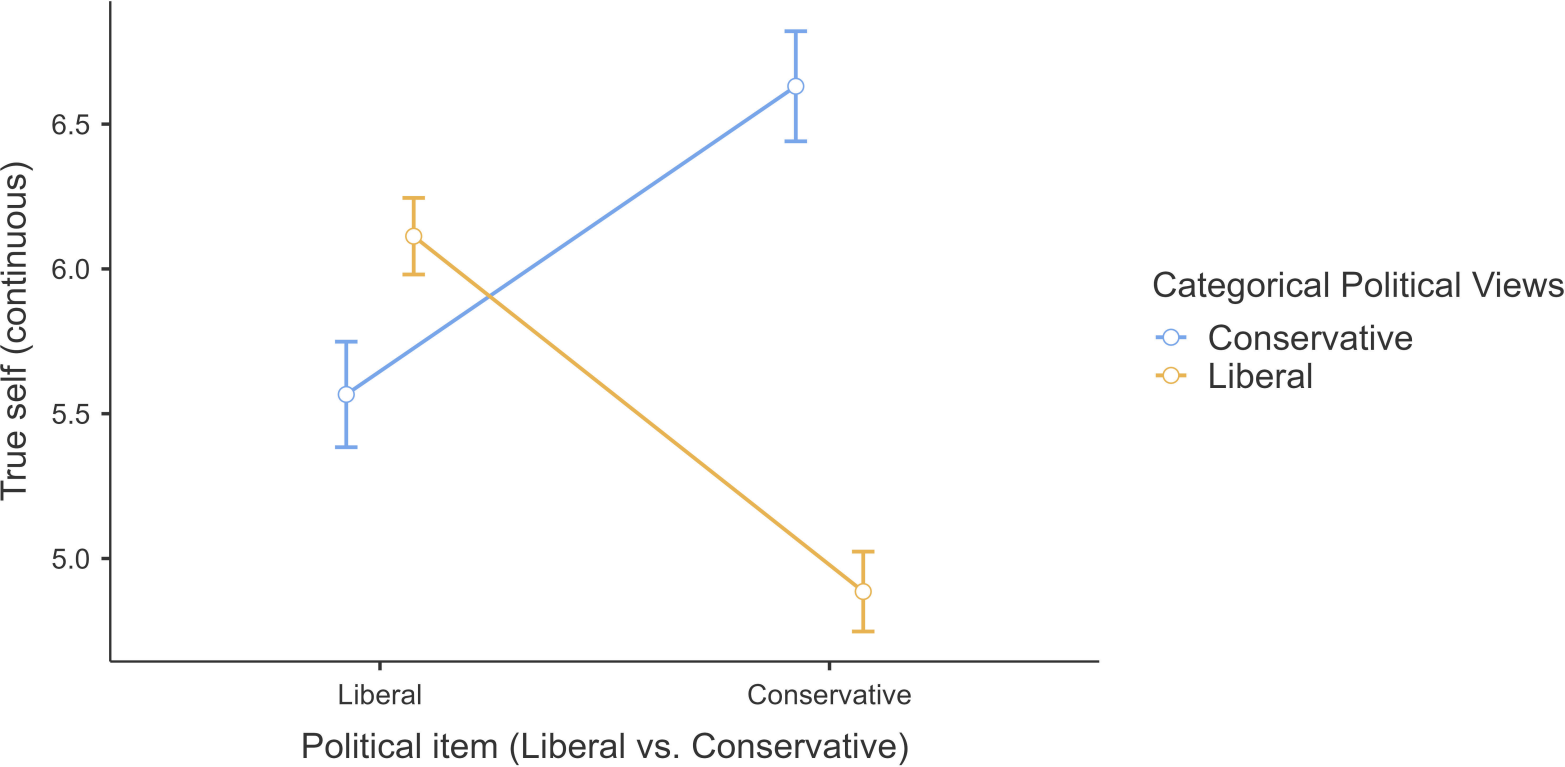
Study 2: interaction between political orientation and political item type on true-self rating. Note. Continuous true-self rating across political items types of liberal and conservative between liberal participants and conservative participants.

Liberal participants were more likely to agree that the behaviour change resulted from the emergence of a person’s true-self for the liberal items (*M* = 6.11, s.d. = 1.39) than for the conservative items (*M* = 4.89, s.d. = 1.48, *t*(630) = 16.84, *p* < 0.001) (figure 3). By contrast, conservative participants were more likely to agree that the behaviour change resulted from the emergence of a person’s true-self for the conservative items (*M* = 5.57, s.d. = 1.32) than for the liberal items (*M* = 6.63, s.d. = 1.33, *t*(630) = −10.60, *p* < 0.001). Similar to the original finding, we also found support for a political views main effect that conservative participants (*M* = 6.10, s.d. = 1.09) tended to rate higher overall true-self ratings than liberal participants (*M* = 5.50, s.d. = 1.24, *t* (630) = 6.03, *p* < 0.001).

Overall, we concluded that we found support for Hypothesis 2 that political views moderated the true-self effect, such that changes more aligned with liberal values were rated as more reflective of true-self by the liberals than the conservatives, whereas changes more aligned with conservative values were rated as more reflective of true-self by the conservatives than the liberals.

### 3.2. Extensions

#### 3.2.1. Study 1: morality valence manipulation check (extension)

We examined whether the participants’ judgement on the different moralized behaviour in Study 1 was aligned with the authors’ hypothesized morality in Study 1, a check that was missing in the target article. We expected that (i) changes categorized as positive would be rated as more positive, and (ii) changes categorized as negative would be rated as more negative.

We summarized descriptive and one-sample *t*‐test results in table 14. We indeed found support for all items being aligned with their assumed valence. We also conducted a series of one-sample *t*-tests on moralized vignettes and found support for the alignment between participants’ and authors’ moral judgement on the vignettes in Study 1.

**Table 14.** Study 1: morality valence manipulation check (one-sample *t*‐test against midpoint 0).

item	good change			bad change		
	*M* (s.d.)	*t*	*p*	*M* (s.d.)	*t*	*p*
alcoholism	67.1 (35.9)	37.7	< 0.001	−51.2 (39.7)	−25.7	< 0.001
boss	76.0 (34.6)	44.4	< 0.001	−73.9 (30.8)	−47.7	< 0.001
parent	83.5 (27.0)	62.5	< 0.001	−75.0 (33.2)	−45.0	< 0.001
terrorism	80.4 (29.7)	53.9	< 0.001	−83.3 (29.5)	−57.0	< 0.001
ethnic minorities	82.8 (26.5)	63.0	< 0.001	−77.7 (34.4)	−44.9	< 0.001
businessman	79.9 (29.1)	54.5	< .001	−75.8 (31.0)	−49.4	< .001
romantic partner	79.9 (27.2)	58.4	< .001	−71.8 (35.7)	−40.6	< .001
police officer	79.7 (31.2)	50.8	< .001	−83.8 (28.0)	−60.5	< .001

	behaviour block 1 (left)			behaviour block 2 (right)		
Mac/PC	3.54 (19.1)			3.73 (18.0)		
country/city	13.99 (29.6)			4.34 (19.1)		
cat/dog	4.60 (17.9)			5.08 (17.4)		
football/baseball	4.13 (18.8)			4.67 (18.7)		

Note. *M* indicates mean. s.d. indicates standard deviation. Scale: −100 to 100, 0 midpoint. *n* was either 408 (block 1) or 395 (block 2), depending on the assigned condition for that item. ‘Behaviour block 1 (left)’ and ‘behaviour block 2 (right)’ refer to sets of neutral items, where block refers to the block of display and right/left refer to which of the pair is displayed. For example, in the Mac/PC pair, Mac = behaviour block 1 (left), PC = behaviour block 2 (right). Valence effects for the positive and negative items were Cohen’s *d* between 1.29 and 3.09.

#### 3.2.2. Study 1: continuous true-self and surface-self measures (extension)

We added two continuous measures inquiring about what the change reflects, one asked about true-self and the other about surface-self.

**Figure 4.**
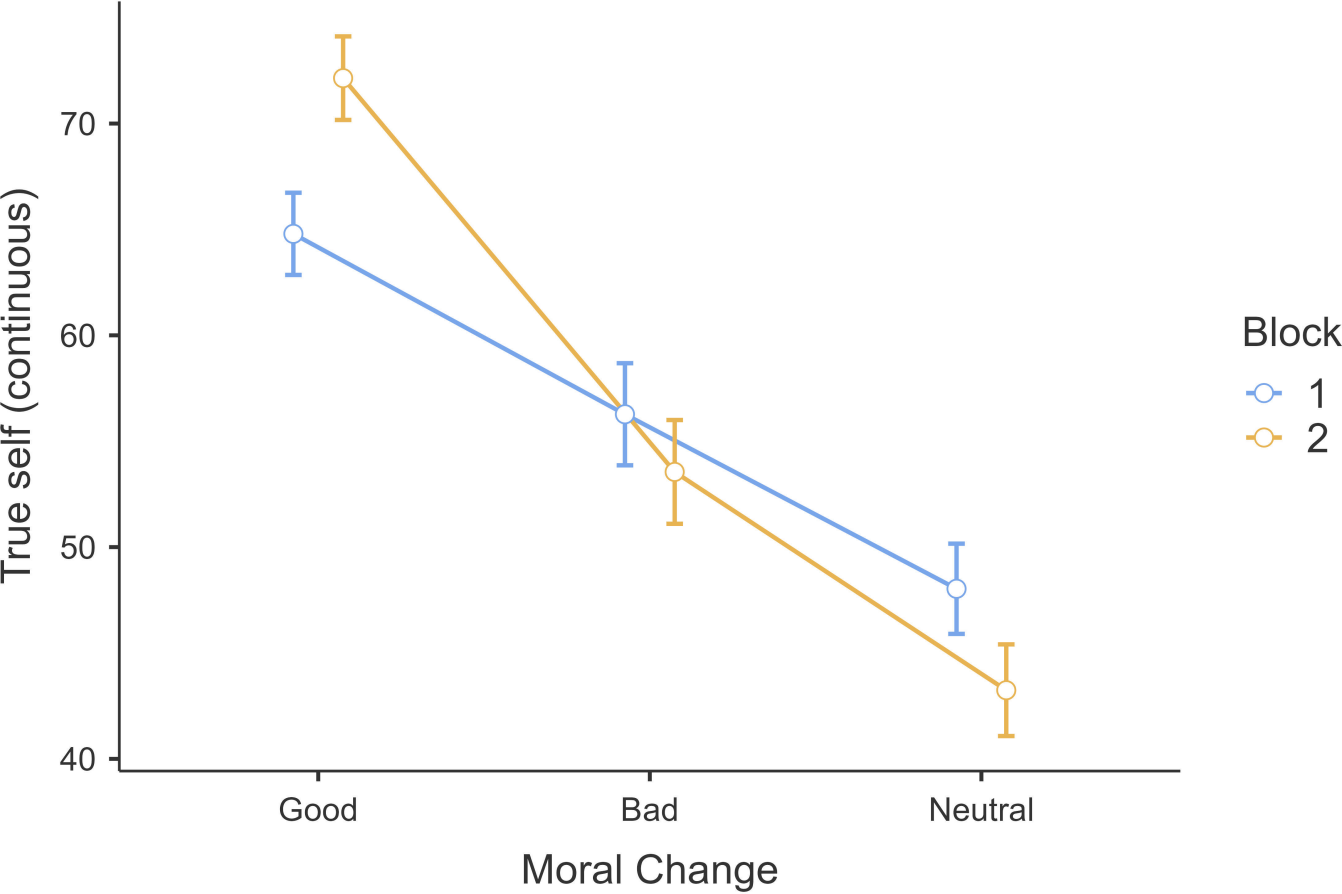
Study 1: continuous true-self measure on moralized changes. Note*.* Continuous true-self measure on good, bad and neutral changes in block 1 and block 2.

Our findings complemented the replication’s continuous true-self after change measure. We first examined the true-self measure and conducted a 3 (moral valence: good, bad, neutral; within) × 2 (order: block 1 and block 2; between) mixed-model ANOVA and found support for a main effect of vignette type (*F*(2, 1602) = 240.5, *p* = 0.001; *η*²*p* = 0.23, 95% CI [0.19, 0.27]) and an interaction effect, but not for block type main effect (see subsection ‘Additional tables and figures’ of electronic supplementary material). We again found support for Hypothesis 1 with a larger effect that participants were more likely to attribute true-self in morally good changes (*M* = 68.4, s.d. = 20.3) than morally bad change (*M* = 54.9, s.d. = 24.8, *t*(801) = 12.27, *p <* 0.001) (figure 4). When compared with neutral change (*M* = 45.7, s.d. = 22.0), true-self was more likely to be revealed in morally good change (*t*(801) = 23.56, *p* < 0.001), and in morally bad change, (*t*(801) = 8.73, *p <* 0*.*001).

**Figure 5.**
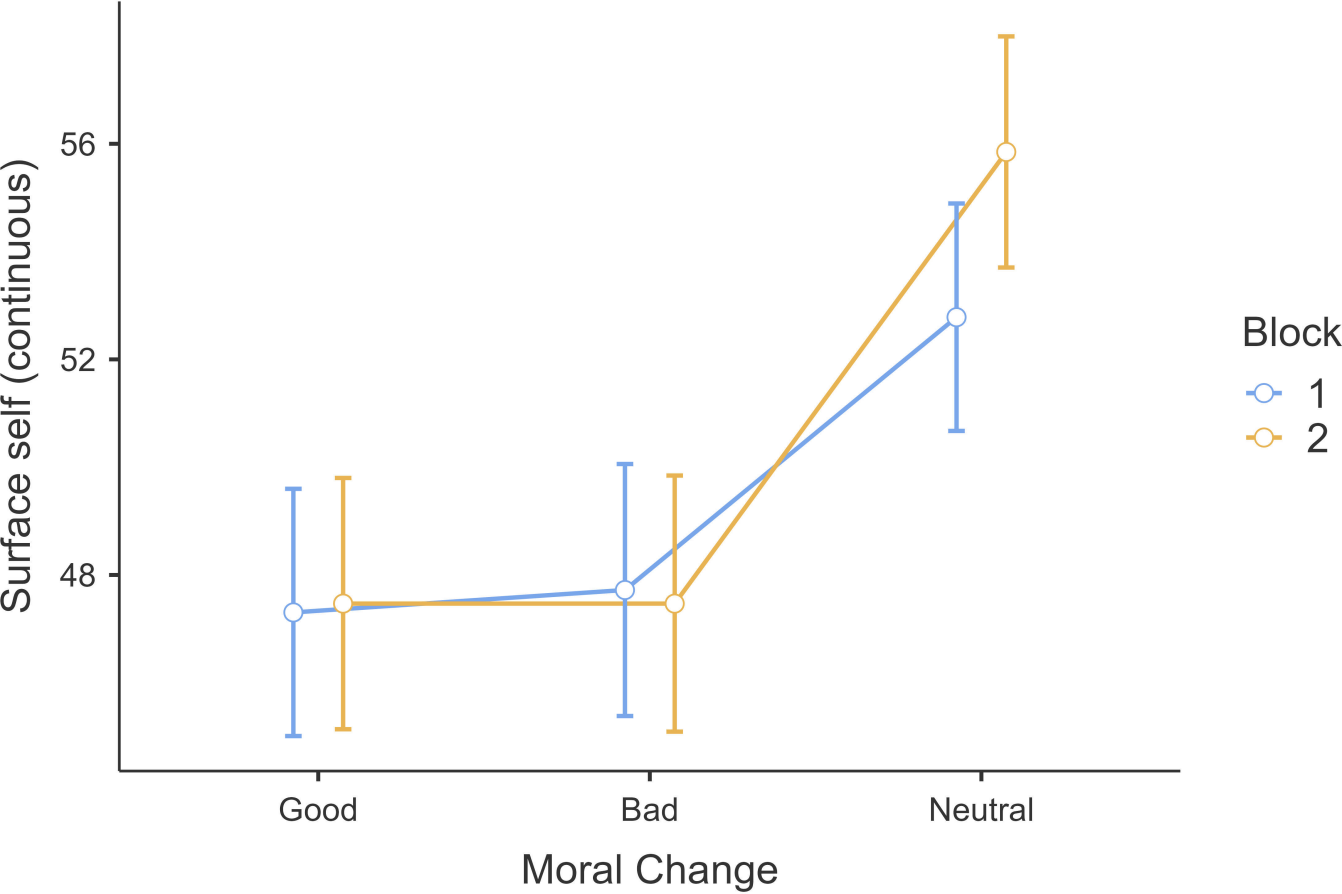
Study 1: continuous surface-self measure on moralized changes. Note. Continuous surface-self measure on good, bad and neutral changes in block 1 and 2.

We conducted a similar test on the surface-self measure using a 3 (moral valence: good, bad, neutral; within) × 2 (order: block 1 and block 2; between) mixed-model ANOVA and found support for a main effect of vignette type (*F*(2,1602) = 36.93, *p* < 0.001; *η*²*p* = 0.04, 95% CI [0.03, 0.06]; details provided in the ‘Additional tables and figures’ subsection of the electronic supplementary material). Yet, we found no support for differences between surface-self ratings for good change (*M =* 47.4, s.d. *=* 23.6) and bad change (*M* = 47.6, s.d. = 24.0, *t*(801) = −0.27, *p* = 0.962; figure 5). Neutral change (*M* = 54.3, Ds.d. = 21.8) was rated as higher surface-self compared with morally good (*t(*801) = −7.17, *p* < 0.001) and morally bad change (*t(*801) = −6.80, *p* < 0.001). We found no support for the hypothesis that surface-self is more reflected in bad change, but rather in neutral changes.

#### 3.2.3. Study 2: vignette political view manipulation check (extension)

We examined whether participants’ judgements of the political views reflected in the changes in the Study 2 vignettes were aligned with the target article authors’ categorizations. We found support for the target’s categorization that indeed all vignettes aligned with the hypothesized political views. We summarized the descriptive and one-sample *t*‐test results for Study 2 in table 15.

**Table 15.** Study 2: vignette political view manipulation check.

item	*M* (s.d.)	*t*	*p*
conservative change			
homosexuality	−35.9 (50.8)	−20.0	< 0.001
patriotism	−41.3 (43.8)	−26.8	< 0.001
theism	−40.1 (45.0)	−25.2	< 0.001
monogamy	−22.1 (39.2)	−16.0	< 0.001
liberal change			
global warming	60.1 (42.2)	40.4	< 0.001
gender equality	56.1 (38.7)	41.1	< 0.001
financial success	34.0 (45.1)	21.4	< 0.001
abortion	50.6 (51.2)	28.0	< 0.001

Note. *M* indicates mean. s.d. indicates standard deviation. *n* = 803. Higher score indicates change is more reflective of liberal rather than conservative political views.

#### 3.2.4. Study 2: interaction between continuous political orientation and political item type on true-self rating (extension)

We added a continuous political orientation scale as an extension. We conducted a 2 (item types: liberal and conservative; within) repeated ANOVA with a continuous covariate of political orientation measure. We found support for the two main effects and the interaction (*F*(1,801) = 409*, p <* 0*.*001, *η*²*p* = .34, 95% CI [0.28, 0.39]) (details provided in subsection ‘Additional tables and figures’ of electronic supplementary material). The findings were consistent with the analysis using the dichotomous political orientation measure. We concluded support for Hypothesis 2 that true-self ratings for change depend on alignment with political views.

#### 3.2.5. Studies 1 and 2: perceived social norms (exploratory)

First, we tested the associations between perceived social norms and morality in Studies 1 and 2. In Study 1, we found support for positive correlations between social norms and all moralized vignettes (most of *r*s ranged from 0.40 to 0.70). In Study 2, for four out of eight politically affiliated vignettes, we found positive correlations between social norms and morality (all *r*s <0.20). We found support for Hypothesis 3a that social norms are positively correlated with morality, especially the positive–negative vignettes.

Second, we tested associations between social norms and true-self attributions. We found support for the positive correlation between social norms and true-self attributions for all the continuous scales (continuous true-self rating and continuous true-self measure in Study 1, and continuous true-self rating in Study 2) (table 16). In Study 1, true-self attributions on the good changes vignettes were positively correlated with norms (all *r*s ranged from 0.07 to 0.21). In Study 2, true-self rating on both liberal and conservative vignettes were positively correlated with norms (all *r*s ranged from 0.10 to 0.30). We found support for the Hypothesis 3a that social norms are positively correlated with true-self attributions.

**Table 16.** Studies 1 and 2: correlation between perceived social norms and overall true-self attributions.

study	true-self attributions	direction of change	*r*	*p*	LCH, HCI
replication					
1	forced-choice measures	positive change	−0.01	0.825	−0.08, 0.06
		negative change	0.02	0.527	−0.01, 0.09
1	continuous true-self rating after change	positive change	0.15	<0.001	0.08, 0.22
		negative change	0.09	0.015	0.02, 0.15
2	continuous true-self rating	liberal change	0.21	<0.001	0.14, 0.27
		conservative change	.16	<.001	.09, .22
extension					
1	continuous true-self measure	positive change	0.13	<0.001	0.07, 0.20
		negative change	−0.03	0.402	−0.10, 0.04
1	continuous surface-self measure	positive change	0.05	0.136	−0.02, 0.12
		negative change	0.07	0.039	0.00, 0.14

Note. LCH and HCI indicate lower confidence intervals and higher confidence intervals, respectively.

#### 3.2.6. Studies 1 and 2: intuitive true-self beliefs (exploratory)

To supplement the indirect way of assessing the link between true-self and morality, we simply asked participants about their intuitions regarding the true-self on the extent to which they thought their and others’ true-self is good and is bad. We ran a 2 (self versus others) × 2 (good versus bad) two-way repeated measures ANOVA, and found support for the main effect for valence (*F*(1,802) = 2888.8, *p* < 0.002, *η*²*p* = 0.73), for main effect of target (*F*(1,802) = 10.0, *p* = 0.002, *η*²*p* = 0.01), and for an interaction (*F*(1,802) = 518.7, *p* < 0.001, *η*²*p* = .39). Ratings of own true-self as good (*M* = 82.8, s.d. = 16.5) were far higher than own true-self as bad (*M* = 15.0, s.d. = 16.9), with a similar yet weaker effect for others (good: *M* = 71.8, s.d. = 17.7; bad: *M* = 27.2, s.d. = 19.6).

We also conducted a correlational analysis exploring the associations between intuitions and true-self attributions in Studies 1 and 2. In table 17, we summarized a comparison of the true-self belief of others and true-self attributions. We found small to moderate correlations with *r*s ranging from 0.08 to 0.28, except the positive true-self belief of others on bad change and negative true-self belief on conservative change. True-self intuitions were positively correlated with true-self attributions. In table 18, we summarized a comparison of the true-self belief of one’s self and true-self attributions. Similarly, we found small to moderate correlations with *r*s ranging from 0.08 to 0.28, except the positive true-self belief of others on bad change and negative true-self belief on conservative change.

**Table 17.** Studies 1 and 2: correlation between the true-self belief of others with the true-self attributions in all vignettes*.*

		true-self intuitions of others: positive			true-self intuitions of others: negative		
study	items	*r*	*p*	LCI,HCI	*r*	*p*	LCI,HCI
1	forced-choice measure (replication)						
	good change	0.12	<0.001	[0.06,0.19]	−0.10	0.007	[−.16, −.03]
	bad change	−0.09	0.009	[−0.16, −0.02]	0.12	<0.001	[0.05,0.19]
1	continuous true-self rating (replication)						
	good change	0.28	<0.001	[0.22,0.34]	−0.28	<0.001	[−0.28,−0.15]
	bad change	−0.13	<0.001	[−0.20,−0.06]	0.14	<0.011	[0.07,0.20]
2	continuous true-self rating (replication)						
	liberal change	0.19	<0.001	[0.12,0.25]	−0.09	0.016	[−0.15,−0.02]
	conservative change	0.05	0.161	[−0.02,0.12]	0.04	0.273	[−0.03,0.11]
1	continuous true-self measure (extension)						
	good change	0.25	<0.001	[0.18,0.31]	−0.16	<0.001	[−0.23,−0.10]
	bad change	−0.03	0.430	[−0.10,0.04]	0.08	0.029	[0.01,0.15]

**Table 18.** Studies 1 and 2: correlation between one’s own true-self belief and the true-self attributions in all vignettes.

		true-self intuitions on self: positive			true-self intuitions in self: negative		
study	items	*r*	*p*	LCI,HCI	*r*	*p*	LCI,HCI
1	forced-choice measure (replication)						
	good change	0.12	<0.001	[0.06,0.19]	−0.10	0.007	[−0.16,−0.03]
	bad change	0.09	0.009	[0.16,0.02]	0.12	<0.001	[0.05,0.19]
1	continuous true-self rating (replication)						
	good change	0.28	<0.001	[0.22,0.35]	0.22	<0.001	[−0.28,−0.15]
	bad change	−0.13	<0.001	[−0.20,−0.06]	−0.16	<0.001	[−0.23,−0.10]
2	continuous true-self rating (replication)						
	liberal change	0.24	<0.001	[0.17,0.30]	−0.13	<0.001	[−0.20,−0.06]
	conservative change	0.17	<0.001	[0.10,0.24]	−0.07	0.053	[−0.14,0.00]
1	continuous true-self measure (extension)						
	good change	0.25	<0.001	[0.18,0.31]	−0.16	<0.001	[−0.23,−0.10]
	bad change	−0.03	0.430	[−0.10,0.04]	0.08	0.029	[0.01,0.15]

### 3.3. Comparison of replication to original findings

We summarize the comparison of the replication and extension in tables 10 and 11. We successfully replicated the results for all the chosen studies with Cohen’s *d* condition comparison effects larger than those reported in the original.

## 4. Discussion

In this Registered Report, we conducted a replication of Newman *et al*. [1]’s Studies 1 and 2 on true-self attributions in value judgement, with added improvements and extensions. We found strong support for all the replication studies and effects.

### 4.1. Replication of Studies 1 and 2

We were successful in replicating the results by Newman *et al*. [1] in support of true-self attribution in value judgement. We found that (i) true-self attributions were higher in positive changes than negative or neutral changes; and (ii) the effect was moderated by political views. Liberals were more likely to view changes towards liberal views as more reflective of true-self than changes towards conservative views, whereas conservatives were more likely to view changes towards conservative views as more reflective of true-self than changes towards liberal views. The findings were consistent across multiple measures, using both forced-choice and continuous scales. There were some minor inconsistencies, as for example we found bad changes were still regarded more as a reflection of true-self than surface-self, yet the pattern of lower true-self ratings and higher surface ratings for negative compared with positive held.

Our findings complement work on true-self in support of true-self perceived as morally good [2,9,12–14,17,34–36]. The diagnostic feature of true-self is rooted in morality, especially positivity. People seem more likely to agree that deep inside humans are good [13]. Some studies suggested that positivity is one of the differences between true-self and self, such that true-self is perceived as good while self can be good or bad [4]. This links with psychological essentialism, which has been used to explain the mechanism behind true-self effect [2,37,38]. Our tendency to perceive the true-self as morally good might be due to the broader tendency to explain things in terms of essences [35].

Moral inferences of negative changes were less certain compared with positive changes, possibly due to the destabilizing impact of bad changes made against societal expectations [39,40]. It is possible that people infer negative changes more cautiously and more diagnostic in updating their views regarding the moral character of an agent [41]. Rather than valence alone, the nature of true-self seems reflective of what individuals value, and changes going counter to values and perceived social norms seem to also go counter to perceived humans’ true-self. True-self might therefore be better described as a dynamic phenomenon taking into account both behaviour and environment rather than focused on the person alone.

### 4.2. Extension: perceived social norms and intuitive true-self belief

In our extension, we found support for the idea that perceived social norms were positively correlated with both morality and true-self attributions. The associations with perceived social norms help bridge the theoretical and methodological shift between the two studies in the target article, the absolute morality depicted in Study 1 with clear positive and negative changes, compared with the relativistic morality depending on political views in Study 2.

Our results aligned with work suggesting that true-self is more strongly reflected in moral changes than in other conventional or personal changes because of the commonly shared nature of morality [17]. Our extensions were preliminary and exploratory, yet our findings suggest that perceived social norms may play a role in true-self attribution, which is somewhat paradoxical, raising the question of how people process the meaning of ‘true’ in ‘true-self’. If laypersons take ‘true’ to mean ‘be yourself’ then this would seem to mean to be about authenticity and staying true to one’s own direction, honouring the expression of one’s self over and possibly against perceived social norms because it highlights the core part of one’s identity. Our findings suggest otherwise, that the morally good behaviour we lead is prone to serve on a pragmatic side of societal consideration instead of a self-enhancing view. The phenomenon of true-self as being moral could be interpreted as serving a functional social role to support socially acceptable behaviours in social interaction, promoting good behaviours for human coexistence to control our urges to act in a socially unacceptable way.

To complement the indirect methods assessing true-self attributions regarding valences and moral changes, we added simplified true-self intuition measures, and found very consistent results, with very large effects for true-self intuitions. People tend to view the self as being far more good than bad, and they consider themselves as more good and less bad than others, with positive associations between true-self intuitions and true-self attributions. The methodology used in the target article may seem overly complex and long, and some of the participants indicated confusion regarding some of the abrupt changes described in the vignettes, which seems to be a limitation in this commonly used paradigm for testing true-self [4]. It is possible that a brief and simple true-self intuitions scale can be used in future research aiming to build on the literature on true-self.

### 4.3. Limitations and future directions

Our study had several limitations. We focused on the replication, with added extensions that were meant to complement the replication and explore new directions for future research. To ensure the replication was unaffected, we only added dependent measures of social norms to allow us to examine associations with true-self attributions, and so our correlational extension findings are only suggestive, and we are unable to infer the causal chain. It seems plausible that true-self attributions are affected by perceptions of whether behaviour is aligned with social norms, yet it is also possible that norms are adjusted in response to true-self perception goals, such as in adjusting perceived social norms to help maintain a more positive self-image. Future research can build on our findings to do additional experimental work to manipulate social norms and examine how they affect true-self attributions, to examine the inherent conflict in whether being ‘true’ is about being different from or in alignment with others.

Second, we tried to follow the original’s materials as closely as possible, yet we noted that we made several adjustments to the original materials and measures to try and debias from issues like gender and ethnicity. We were successful in our replications, yet it is difficult to estimate how much our changes have impacted the results. Some issues remained unaddressed, also raised in our review process. For example, the forced-choice measure of true-self in Study 1 might be further improved, as to not force the experimenter’s understanding of true- and surface-self onto the participants. For example, the original question read ‘This person’s “surface-self” (the things this person learned from society or others)’ seems to explicitly imply that surface-self reflects learned thoughts or behaviours that are different from the true-self. Yet, our findings with other items seem to suggest that such a description of ‘surface-self’ might not always be aligned with how people think of true- and surface-self, raising the possibility that it is actually true-self that is aligned with society and others, not (only) the surface-self. Reviewer Dr. A. G. Christy suggested that laypersons are unfamiliar with the term ‘essential’ or ‘non-essential’ and that the use of this terminology might further bias responses. Therefore, we suggest future research to carefully rethink the way that true- and surface-selves are described to get closer to what we aim to study—people’s lay-perceptions. In this specific example, future studies may consider changing the descriptions to ‘This person’s true-self (the deepest, most core aspect of this person’s being)’ versus ‘This person’s surface-self (the shallowest, and more peripheral aspect of this person’s being)’ or simply referring to ‘true-self’ and ‘surface-self’ and letting people infer from that what they will.

In our initial submission we raised concerns regarding the methodological choice in Study 1 to fix the display of items so that each block first displays four positive (/negative) changes together and then four negative (/positive) change vignettes together, followed by four neutral vignettes, which the original authors explained as contrasting certain changes against each other. There were some minor block-order effects that did not seem to impact the overall pattern of results, yet in future research it might be better to randomize the display of the vignettes within each block.

Finally, some of our participants (in the feedback section) and one of our reviewers, Dr. Sergio Barbosa, expressed concern that the current vignettes did not make any reference to a ‘mind’ behind the described actions. Some participants reflected that there was insufficient information, like the motivation behind changes, to be able to evaluate the true-self of the agent for that behaviour. The daily value judgement would be different from the fictional change used in the study. It could be more complicated because the judgement on changes might involve other considerations like personal development [42]. From a recent review, moral judgement is not centred on the behaviour but could be a summary judgement including but not restricted to the mind of the agent such as intention, explanations and capacities or even the perceived strength of the agent [43]. Thus, future research can build on these findings to further explore the role of intent in attributions of true-self.

## Data Availability

We provided all materials, data and code on: [44]. Supplementary material is available online [45].

## References

[1] Newman GE, Bloom P, Knobe J. 2014 Value judgments and the true self. Personal. Soc. Psychol. Bull. **40**, 203–216. (10.1177/0146167213508791) 24154918

[2] De Freitas J, Cikara M, Grossmann I, Schlegel R. 2017 Origins of the belief in good true selves. Trends Cogn. Sci. **21**, 634–636. (10.1016/j.tics.2017.05.009) 28601535

[3] Schlegel RJ, Hicks JA, King LA, Arndt J. 2011 Feeling like you know who you are: perceived true self-knowledge and meaning in life. Personal. Soc. Psychol. Bull. **37**, 745–756. (10.1177/0146167211400424) 21402753

[4] Strohminger N, Knobe J, Newman G. 2017 The true self: a psychological concept distinct from the self. Perspect. Psychol. Sci. **12**, 551–560. (10.1177/1745691616689495) 28671854

[5] Newman GE, De Freitas J, Knobe J. 2015 Beliefs about the true self explain asymmetries based on moral judgment. Cogn. Sci. **39**, 96–125. (10.1111/cogs.12134) 25039306

[6] Johnson JT, Robinson MD, Mitchell EB. 2004 Inferences about the authentic self: when do actions say more than mental states? J. Personal. Soc. Psychol. **87**, 615. (10.1037/0022-3514.87.5.615) 15535775

[7] Newman GE, Knobe J. 2019 The essence of essentialism. Mind Lang. **34**, 585–605. (10.1111/mila.12226)

[8] Schlegel RJ, Hicks JA, Arndt J, King LA. 2009 Thine own self: true self-concept accessibility and meaning in life. J. Personal. Soc. Psychol. **96**, 473–490. (10.1037/a0014060) PMC4714566 19159144

[9] Strohminger N, Nichols S. 2014 The essential moral self. Cognition **131**, 159–171. (10.1016/j.cognition.2013.12.005) 24503450

[10] Wong C, Feldman G. 2019 Essential moral self remains unchallenged: successful replication of Strohminger and Nichols (2014) with extensions comparing morality to ideology and religion. OSF. (10.17605/OSF.IO/G2ZV6)

[11] Kumar V. 2016 The empirical identity of moral judgment: table 1. Philos. Q. **66**, 783–804. (10.1093/pq/pqw019)

[12] De Freitas J, Sarkissian H, Newman GE, Grossmann I, De Brigard F, Luco A, Knobe J. 2018 Consistent belief in a good true self in misanthropes and three interdependent cultures. Cogn. Sci. **42**, 134–160. (10.1111/cogs.12505) 28585702

[13] Heiphetz L, Strohminger N, Young LL. 2017 The role of moral beliefs, memories, and preferences in representations of identity. Cogn. Sci. **41**, 744–767. (10.1016/j.jesp.2018.03.007) 26936631

[14] Heiphetz L. 2019 Moral essentialism and generosity among children and adults. J. Exp. Psychol. **148**, 2077–2090. (10.1037/xge0000587) 30829523

[15] Nosek BA *et al*. 2022 Replicability, robustness, and reproducibility in psychological science. Annu. Rev. Psychol. **73**, 719–748. (10.1146/annurev-psych-020821-114157) 34665669

[16] Zwaan RA, Etz A, Lucas RE, Donnellan MB. 2018 Making replication mainstream. Behav. Brain Sci. **41**, e120. (10.1017/s0140525x17001972) 29065933

[17] Lefebvre JP, Krettenauer T. 2020 Is the true self truly moral? Identity intuitions across domains of sociomoral reasoning and age. J. Exp. Child Psychol. **192**, 104769. (10.1016/j.jecp.2019.104769) 31931394

[18] Chan M, Feldman G. 2025 Factors impacting effective altruism: revisiting heuristics and biases in charity in a replication and extension Registered Report of Baron and Szymanska (2011). R. Soc. Open Sci. (10.17605/OSF.IO/BEP78) PMC12092110 40400516

[19] Ding K, Feldman G. 2025 Revisiting partition priming in judgment under uncertainty: replication and extension Registered Report of Fox and Rottenstreich (2003). R. Soc. Open Sci. (10.17605/OSF.IO/G9CZS) PMC12308232 40740696

[20] Wong CL, Feldman G. 2025 Choice bracketing revisited: replication and extensions Registered Report of seven experiments reviewed in Read et al. (1999). R. Soc. Open Sci. (10.17605/OSF.IO/VDQEK) PMC11964406 40177105

[21] Graham J, Haidt J, Nosek BA. 2009 Liberals and conservatives rely on different sets of moral foundations. J. Personal. Soc. Psychol. **96**, 1029–1046. (10.1037/a0015141) 19379034

[22] Feldman G. 2017 Making sense of agency: belief in free will as a unique and important construct. Soc. Personal. Psychol. Compass **11**, e12293. (10.1111/spc3.12293)

[23] Feldman G, Chandrashekar SP. 2018 Laypersons’ beliefs and intuitions about free will and determinism: new insights linking the social psychology and experimental philosophy paradigms. Soc. Psychol. Personal. Sci. **9**, 539–549. (10.1177/1948550617713254) 30220960 PMC6113710

[24] Chambers C. 2025 Successfully replicating positive evaluations of our ‘true selves’*.* Peer Community in Registered Reports. (10.24072/pci.rr.100372)

[25] CORE Team. 2025 Collaborative open-science and meta research. OSF. http://osf.io/5z4a8

[26] Feldman G. 2023 Registered Report Stage 1 manuscript template. OSF. (10.17605/OSF.IO/YQXTP)

[27] Jané M *et al*. 2024 Guide to effect sizes and confidence intervals. OSF (10.17605/OSF.IO/D8C4G)

[28] Simonsohn U. 2015 Small telescopes: detectability and the evaluation of replication results. Psychol. Sci. **26**, 559–569. (10.1177/0956797614567341) 25800521

[29] Faul F, Erdfelder E, Lang AG, Buchner A. 2007 G*Power 3: a flexible statistical power analysis program for the social, behavioral, and biomedical sciences. Behav. Res. Methods **39**, 175–191. (10.3758/bf03193146) 17695343

[30] Litman L, Robinson J, Abberbock T. 2017 TurkPrime.com: a versatile crowdsourcing data acquisition platform for the behavioral sciences. Behav. Res. Methods **49**, 433–442. (10.3758/s13428-016-0727-z) 27071389 PMC5405057

[31] LeBel EP, McCarthy RJ, Earp BD, Elson M, Vanpaemel W. 2018 A unified framework to quantify the credibility of scientific findings. Adv. Methods Pract. Psychol. Sci. **1**, 389–402. (10.1177/2515245918787489)

[32] LeBel EP, Vanpaemel W, Cheung I, Campbell L. 2019 A brief guide to evaluate replications. Meta Psychol. **3**, 1–9. (10.15626/mp.2018.843)

[33] JAMOVI project. 2023 jamovi (Version 2.4). Computer software. See https://www.jamovi.org.

[34] Christy AG, Seto E, Schlegel RJ, Vess M, Hicks JA. 2016 Straying from the righteous path and from ourselves: the interplay between perceptions of morality and self-knowledge. Personal. Soc. Psychol. Bull. **42**, 1538–1550. (10.1177/0146167216665095) 27655752

[35] Christy AG, Kim J, Vess M, Schlegel RJ, Hicks JA. 2017 The reciprocal relationship between perceptions of moral goodness and knowledge of others’ true selves. Soc. Psychol. Personal. Sci. **8**, 910–917. (10.1177/1948550617693061)

[36] Fernandez-Duque D, Schwartz B. 2016 Common sense beliefs about the central self, moral character, and the brain. Front. Psychol. **6**, 2007. (10.3389/fpsyg.2015.02007) 26793140 PMC4709419

[37] Haslam N, Bastian B, Bissett M. 2004 Essentialist beliefs about personality and their implications. Personal. Soc. Psychol. Bull. **30**, 1661–1673. (10.1177/0146167204271182) 15536247

[38] Neufeld E. 2022 Psychological essentialism and the structure of concepts. Philos. Compass **17**, e12823. (10.1111/phc3.12823)

[39] Siegel J, Mathys C, Rutledge R, Crockett M. 2018 Beliefs about bad people are volatile. Nat. Hum. Behav. **2**, 750–756. (10.31234/osf.io/2cqkz) 31406285

[40] Siedlecki P, Baron SG, Todorov A. 2013 Diagnostic value underlies asymmetric updating of impressions in the morality and ability domains. J. Neurosci. **33**, 19406–19415. (10.1523/jneurosci.2334-13.2013) 24336707 PMC6618766

[41] Klein N, O’Brien E. 2016 The tipping point of moral change: when do good and bad acts make good and bad actors? Soc. Cogn. **34**, 149–166. (10.1521/soco.2016.34.2.149)

[42] Molouki S, Bartels DM. 2017 Personal change and the continuity of the self. Cogn. Psychol. **93**, 1–17. (10.1016/j.cogpsych.2016.11.006) 28039761

[43] Hartman R, Blakey W, Gray K. 2022 Deconstructing moral character judgments. Curr. Opin. Psychol. **43**, 2021. (10.1016/j.copsyc.2021.07.008) 34418790

[44] Lee SC, Feldman G. 2025 Revisiting the link between true-self and morality: Replication and extensions of Newman, Bloom and Knobe (2014) Studies 1 and 2. OSF. 10.17605/OSF.IO/9FVTQ PMC12187398 40568552

[45] Lee SC, Feldman G. 2025 Supplementary material from: Revisiting the link between true-self and morality: Replication and extension Registered Report of Newman, Bloom, and Knobe (2014) Studies 1 and 2. Figshare. (10.6084/m9.figshare.c.7837943) PMC12187398 40568552

